# Toward standardized epitranscriptome analytics: an inter-laboratory comparison of mass spectrometric detection and quantification of modified ribonucleosides in human RNA

**DOI:** 10.1093/nar/gkaf895

**Published:** 2025-09-12

**Authors:** Martin Hengesbach, Chi-Kong Chan, Tulsi Bhandari, Alan Bruzel, Michael S DeMott, Ganna Podoprygorina, Guangxin Sun, Ellen Tabeling, Vivian G Cheung, Peter C Dedon, Mark Helm, Patrick A Limbach

**Affiliations:** Institute for Pharmaceutical and Biomedical Sciences, Johannes Gutenberg University, Mainz, 55128, Germany; Department of Biological Engineering, Massachusetts Institute of Technology, Cambridge, MA 02139, United States; Department of Chemistry, Rieveschl Laboratories for Mass Spectrometry, University of Cincinnati, Cincinnati, OH 45221, United States; Department of Pediatrics, University of Michigan School of Medicine, Ann Arbor, MI 48109, United States; Departments of Molecular Biology, Cell Biology and Biochemistry and Pediatrics, Brown University, Providence, RI 02903, United States; Institute for Pharmaceutical and Biomedical Sciences, Johannes Gutenberg University, Mainz, 55128, Germany; Department of Biological Engineering, Massachusetts Institute of Technology, Cambridge, MA 02139, United States; Department of Chemistry, Rieveschl Laboratories for Mass Spectrometry, University of Cincinnati, Cincinnati, OH 45221, United States; Department of Pediatrics, University of Michigan School of Medicine, Ann Arbor, MI 48109, United States; Department of Biological Engineering, Massachusetts Institute of Technology, Cambridge, MA 02139, United States; Institute for Pharmaceutical and Biomedical Sciences, Johannes Gutenberg University, Mainz, 55128, Germany; Department of Chemistry, Rieveschl Laboratories for Mass Spectrometry, University of Cincinnati, Cincinnati, OH 45221, United States

## Abstract

The human RNome comprises all forms of RNA and the 50 + chemical structures—the epitranscriptome—that modify them. Understanding the diverse functions of RNA modifications in regulating gene expression and cell phenotype requires technologies such as RNA sequencing-based modification mapping and mass spectrometry-based quantification of modified ribonucleosides. Liquid chromatography-coupled tandem quadrupole mass spectrometry (LC–MS/MS) is the gold standard for detecting and quantifying modified ribonucleosides with accuracy and precision. However, variations in RNA isolation, processing, and LC–MS/MS analysis have hindered reproducibility across laboratories, which is essential for accurate quantification of RNA modifications. As guidance toward harmonization, we report a multi-laboratory comparison of workflows for LC–MS/MS RNA modification analysis. We compared protocols for sample shipment, RNA hydrolysis, LC–MS/MS analysis, and data processing among three laboratories working with the same total RNA samples. We detected and quantified 17 modifications consistently across protocols and operators, with another 7 that were sensitive to experimental conditions, reagent contamination, and ribonucleoside instability, leading to poor precision among laboratories. Agreement among the three labs was strong, with coefficients of variation of 20% and 10% for relative and absolute quantification, respectively. These findings establish a robust and readily adoptable epitranscriptome analytical platform that enables reliable comparisons across laboratories.

## Graphical Abstract

**Graphical Abstract:**
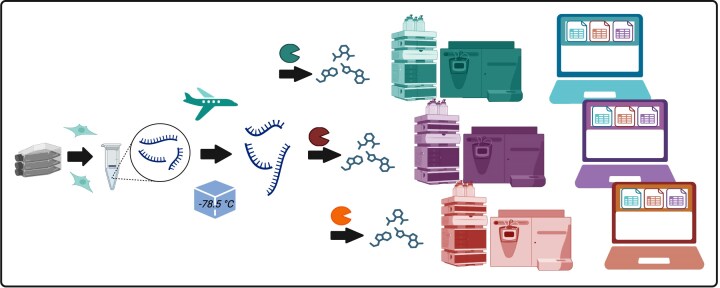

## Introduction

Of all macromolecules with post-synthetic modifications, RNA ranks among the most extensively modified, with more than 170 post-transcriptional modifications comprising the epitranscriptome [1] and the complete set of all RNA molecules and their modifications comprising the RNome [2]. The number of possible modifications is far from fully defined, with new ones being discovered on a frequent basis [3]. The complexity of the epitranscriptome is illustrated by the fact that many modifications are ubiquitous across all kingdoms of life, while some are highly unique to specific niches.[1] As post-transcriptional modifications can be installed by hundreds of conserved enzymes, their location and abundance in specific RNA species regulate numerous aspects of cell physiology and survival, most notably in regulating gene expression at the level of translation. Dysregulation of RNA modification is now recognized as the basis for nearly 100 human diseases [4, 5]. The immensity of the scope and impact of the epitranscriptome on human health has raised concerns about the lack of validated and accessible technologies for quantifying all RNA modifications and mapping them in all RNA molecules. A recent report from the National Academies of Science, Engineering, and Medicine, “Charting a Future for Sequencing RNA and Its Modifications,” highlights these problems and the need for robust technologies [2].

Analysis of the quantities and locations of modifications in the RNome can be approached with several orthogonal techniques. Direct and next-generation sequencing-based approaches allow for site-specific and sometimes quantitative determination of many RNA modifications [6]. For example, Nanopore-based direct RNA sequencing, which is based on indirect methods of detection, can so far identify the presence of a handful of modifications along an RNA sequence, with limited ability to accurately identify a specific modification without prior knowledge and missing information at the ends of the RNA molecules [7–9]. Mass spectrometry (MS), which provides molecular mass and structural information, allows accurate and precise identification and quantification of all types of modified ribonucleosides [10–12]. As a complement to the long reads achieved with Nanopore sequencing, high-resolution tandem MS methods have been developed to site-specifically identify and quantify modifications in oligonucleotides up to 25 nt in length [13], though the technology is in its infancy. Prior to and as a complement to sequencing-based modification mapping, it is critical to know which modifications are present in an organism or RNA sample. Indeed, changes in the levels of the dozens of modifications on transfer RNA molecules (tRNA) are highly predictive of cell response to environmental stressors [14]. As the gold standard for detecting and quantifying all modified ribonucleosides in a sample with accuracy and precision, liquid chromatography-coupled tandem quadrupole MS (LC–MS/MS) technology allows concomitant analysis of nearly all types of modifications in a single RNA sample without regard to sequence context [10–12].

Despite the utility of LC–MS/MS for detecting and quantifying modified ribonucleosides, there has been no attempt to create a standardized protocol that ensures rigorous and reproducible results across the full experimental scope, from RNA purification to processing of the output data from the mass spectrometer. Indeed, a recent comprehensive review highlights the many artefacts and pitfalls reported in LC–MS/MS analysis of RNA modifications [15]. Starting at the first step of purification, RNA is among the most unstable biomolecules, well known to undergo hydrolysis at extremes of pH and degradation by contaminating nucleases [15–17], which raises concerns about RNA integrity during purification and shipment. Several types of RNA modification are also chemically unstable, including conversion of 1-methyladenosine (m^1^A) to *N*^6^-methyladenosine (m^6^A) by the Dimroth rearrangement [18], hydrolytic ring-opening of cyclic N^6^-threonylcarbamoyladenosine (ct^6^A), oxidation of 5-hydroxyuridine (ho^5^U) and 5-hydroxycytidine (ho^5^C) [19], and hydrolysis of *N*^4^-acetylcytidine (ac^4^C) [20]. Some modifications are prone to artifactual formation, such as deamination of adenosine (A) to inosine (I) by enzymes present during RNA isolation and contaminating commercial phosphatases [21]. The method of RNA purification itself can also introduce artifacts, such as phenol extraction employing guanidinium thiocyanate [22] noted to be biased against short G/C-rich RNA sequences, and purification methods introducing rRNA fragments into small RNA fractions (miRNA, tRNA, etc.) and vice versa [23].

For the next step of RNA processing for LC–MS/MS analysis, there are many published methods that use nonspecific nucleases (e.g. nuclease P1, nuclease S1, benzonase) to cleave RNA into 5′-monophosphoribonucleotides, which are further dephosphorylated to ribonucleosides with a phosphatase (e.g. calf intestinal, shrimp, heat-labile alkaline) [12, 24, 25]. However, these processing methods have not been rigorously compared for quantitative accuracy. In addition to the artefacts noted for RNA purification, there are a variety of problems caused by carryover of buffer components to the LC–MS/MS system [26] and loss of specific modified ribonucleosides on centrifugal filters and other processing devices [15].

In LC–MS/MS analysis step, the hydrolyzed ribonucleosides in the RNA sample are resolved by reversed-phase chromatography before being ionized, fragmented, and detected in the MS system. Here, again, there are numerous potential problems that can affect the accuracy, precision, and sensitivity of the analysis, including differences among the many different types of reversed-phase HPLC columns and MS/MS instruments available. The large variety of C18 reversed-phase HPLC columns, the most commonly used separation system for modified ribonucleosides, differ significantly in the order of elution of ribonucleosides and resolving power, which can present significant problems. For example, co-elution or close elution of isobaric positional isomers (e.g. 1-methylguanosine, m^1^G, and *N*^2^-methylguanosine, m^2^G; or 2-methyladenosine, m^2^A, and m^6^A) can lead to errors in accurate quantification, while signals for rare ribonucleosides can be suppressed by co-elution with the highly abundant canonical ribonucleosides (adenosine, A; guanosine, G; cytidine, C; uridine, U) [12, 24, 25]. Similarly, inosine (I, 268 Da) and the + 1 Da isotopomer (268 Da) of the highly abundant A (267 Da) can be confused if the two ribonucleosides elute close together. Further complicating the situation is the fact that the machine-generated signal does not necessarily correlate with the abundance of a ribonucleoside. MS/MS instruments vary in terms of the efficiency of ion generation, focusing, transfer, accumulation, fragmentation, and finally detection for each ribonucleoside and product ion. The chemical structure of the ribonucleoside also affects the detected signal, with lower ionization efficiencies for pyrimidine-based modifications than those of purines [27]. While relative quantification of a ribonucleoside across multiple samples can provide highly precise analyses and absolute quantification can be achieved with internal standards or calibration against external standards [12, 24, 25], variation in the amount of the hydrolyzed RNA sample injected onto the HPLC column must be controlled. This is most readily achieved by quantifying the signal intensities for some or all the canonical ribonucleosides, using either the MS signals or the UV absorbance value from an in-line detector [12, 24, 25]. Compared to modified ribonucleosides, canonical ribonucleosides are typically found in quantities that are many orders of magnitude higher in the RNA hydrolysate, making it easy to saturate the highly sensitive MS detectors or suppress signals for rare co-eluting ribonucleosides [12, 24, 25].

Coupled with the growing interest in RNA modifications and the RNome [2], all of these problems highlight the importance of developing standardized protocols for all RNA-directed analytical methods, including LC–MS/MS, to minimize the many sources of variation and maximize precision and accuracy at all steps from RNA isolation to processing the data output from the MS. Here we report an effort by four laboratories to compare and in some cases optimize LC–MS/MS RNA modification analysis. Using a common RNA sample, we focused on four major tasks involved in performing LC–MS/MS analysis of modified ribonucleosides: RNA preparation and shipping, RNA hydrolysis, LC–MS/MS analysis, and data processing. We identified modifications that were consistently detected and quantified with high precision across the laboratories, as well as modifications that were sensitive to experimental conditions, reagent contamination, and ribonucleoside instability. The results provide a robust, transferable platform for the analysis of modified ribonucleosides, which minimizes many sources of error to allow reliable comparisons among different research groups studying the RNome and can be adopted by any LC–MS/MS-competent research group. Like the standardization of DNA microarray technology 25 years ago [28, 29], this inter-laboratory comparison of epitranscriptome analytical platforms ensures precision, accuracy, and rigor among researchers and core facilities around the world.

## Materials and methods

**Figure 1.**
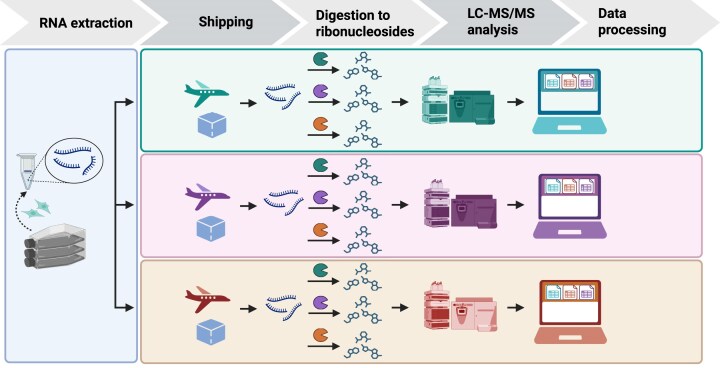
An inter-laboratory study to identify robust methods for all steps in the quantitative analysis of RNA modifications: RNA shipment, RNA digestion, LC–MS/MS analysis of RNA modifications, and data processing. Created in BioRender. Dedon, P. (2025) https://BioRender.com/w90dhut.

As depicted in Fig. 1, this study was conducted by four laboratories with the goal of identifying robust methods for preparing and shipping RNA, hydrolyzing the RNA to component ribonucleosides, analysis of the ribonucleosides by LC–MS/MS, and processing of the resulting data. RNA samples were prepared by the Cheung Lab at University of Michigan (UM), USA. The samples were then shipped to the Helm Lab at Johannes Gutenberg University (JGU), Germany, the Dedon Lab at the Massachusetts Institute of Technology (MIT), USA, and the Limbach Lab at the University of Cincinnati (UC), USA, for processing and analysis of RNA modifications by LC–MS/MS. Materials and methods used in each laboratory are described in detail below and in Supplementary Tables S1 and S2.

### Reagents

All chemicals and reagents used in this study are listed in Supplementary Table S1 with sources noted in Germany and the USA. They were of the highest purity obtainable and used without further purification. Deionized water was further purified with laboratory water purification systems and used in LC–MS/MS analysis.

### Cell culture and total RNA extraction at UM

Human embryonic kidney 293 (HEK293) cells were obtained from American Type Culture Collection (#CRL-1573; Manassas, VA, USA). Cells (∼2 × 10^6^) were cultured in Dulbecco's modified Eagle’s medium supplemented with 10% fetal bovine serum, 2 mM L-glutamine, and 1% penicillin-streptomycin in a humidified incubator kept at 5% CO_2_ and 37°C. Upon reaching 100% confluency, cells were gently rinsed with pre-warmed PBS, detached with trypsin-EDTA at 37°C for 5 min, and pelleted by centrifugation at 125 x g at 4°C for 5 min. Cell pellets (∼3 × 10^7^ cells) were resuspended in 2 mL of Buffer RLT containing 0.14 M 2-mercaptoethanol and vortexed for 30 s for complete homogenization. The lysate was passed through an 18-gauge needle 10 times, mixed thoroughly with 1 volume of 70% ethanol, and loaded onto a RNeasy Midi column. Total RNA was then extracted following the manufacturer’s protocol and eluted from the column in two washes of 250 μL diethylpyrocarbonate (DEPC)-treated water (ThermoFisher #AM9915G; autoclaved by the manufacturer to remove residual DEPC). RNA concentration and integrity were assessed using a Qubit RNA High Sensitivity Assay Kit (ThermoFisher #Q32852) and an Agilent 2100 Bioanalyzer (Agilent Technologies, Santa Clara, CA, USA), respectively. The RNA eluates were then aliquoted and stored at –80°C prior to further processing.

### RNA shipment preparation

To evaluate the effect of shipping conditions on RNA integrity, the purified total RNA samples were processed at UM for shipping under four conditions: (1) shipment of RNA in aqueous ethanol (EtOH): RNA samples were adjusted to 0.5 M ammonium acetate (NH_4_OAc) and brought to 75% EtOH; (2) shipment of RNA pelleted by EtOH precipitation and dried: RNA samples were adjusted to 0.5 M NH_4_OAc and brought to 75% EtOH using ice-cold absolute EtOH, followed by 30 s vortexing and 30 min centrifugation at 12 500 x g at 4°C. Pellets were washed twice with 75% EtOH and air-dried before shipping; (3) shipment of RNA pelleted by lithium chloride (LiCl)/EtOH precipitation and dried: RNA samples were adjusted to 0.8 M LiCl and brought to 75% EtOH using ice-cold absolute EtOH, followed by 30 s vortexing and 30 min centrifugation at 12 500 x *g* at 4°C. Pellets were washed twice with 75% EtOH and air-dried before shipping, and (4) shipment of RNA in DPEC-treated water: RNA samples were directly shipped after elution from RNA extraction columns. The processed RNA samples were then shipped at ambient temperature or on dry ice to the three analytical laboratories: JGU, MIT, and UC.

### Instrumentation

RNA integrity was assessed upon arrival at each lab using an Agilent 2100 Bioanalyzer (MIT, UC) or an Agilent 4200 TapeStation (JGU) (both Agilent Technologies, Santa Clara, CA, USA). Agilent RNA 6000 Nano Kits or RNA 6000 Pico Kits (for Bioanalyzer) and Agilent High Sensitivity RNA ScreenTape (for TapeStation) were used to analyze RNA with sizes ranging from 50 to 6000 nt. The RNA Integrity Number (RIN) was used as a metric for sample quality.

RNA modification analysis was performed using three different LC–MS/MS systems. JGU: an Agilent 1260 high-performance liquid chromatography (HPLC) system equipped with an inline diode array detector (DAD) was coupled to an Agilent 6460 triple quadrupole mass spectrometer (Agilent Technologies, Santa Clara, CA, USA). MIT: an Agilent 1290 HPLC system equipped with an inline DAD was coupled to an Agilent 6495c triple quadrupole mass spectrometer (Agilent Technologies, Santa Clara, CA, USA). UC: a Vanquish ultrahigh-performance liquid chromatography (UHPLC) system was coupled to a TSQ Quantiva triple-stage quadrupole mass spectrometer (Thermo Fisher Scientific Inc., San Jose, CA, USA).

### RNA hydrolysis

Using RNA samples with RIN scores of 8–10, the RNA was processed for LC–MS/MS analysis by enzymatic hydrolysis and dephosphorylation to ribonucleosides. Hydrolysis was conducted with protocols previously established in the three participating laboratories as follows. JGU: for ≤10 μg of total RNA, reactions (40 μL) containing 5 mM Tris (pH 8), 1 mM magnesium chloride (MgCl_2_), 200 ng of pentostatin (adenosine deaminase inhibitor), 10 U benzonase, 0.6 U nuclease P1, 0.2 U bovine intestinal alkaline phosphatase, 0.2 U snake venom phosphodiesterase, and filtered water were incubated at 37°C for 2 h. The hydrolyzed samples were either analyzed immediately or stored at –20°C until analysis. MIT: for ≤5 μg of total RNA, reactions (50 μL) containing 5 mM Tris (pH 8), 2.5 mM MgCl_2_, 5 ng of coformycin (adenosine deaminase inhibitor), 8 U benzonase, 4.9 U calf intestinal alkaline phosphatase, 0.15 U snake venom phosphodiesterase, 0.1 mM deferoxamine (DFOA, antioxidant), 0.1 mM butylated hydroxytoluene (BHT, antioxidant), 50 nM [^15^N_5_]-2′-deoxyadenosine (MIT internal standard), 1.25 ng of 5-bromocytidine (5-BrC; internal standard for quality control for all labs), and RNase-free water were incubated at 37°C for 6 h. The hydrolysate was either analyzed immediately, stored at 4°C for ≤16 h, or stored at −80°C. UC: for ≤4 μg of total RNA, reactions (20 μL) containing 24.8 mM NH_4_OAc (pH 5), 22 μM zinc chloride, 0.3 U nuclease P1, 0.1 U snake venom phosphodiesterase, 2 ng of 5-BrC, and autoclaved milliQ water were incubated at 37°C for 2 h. FastAP thermosensitive alkaline phosphatase (1 U) and 2.4 μL FastAP reaction buffer were then added with incubation at 37°C for 1 h. After incubation, the digested samples were dried with a SpeedVac concentrator and redissolved in mobile phase A. Samples were either analyzed immediately, stored at -20°C for short-term storage, or stored at −80°C. We encourage readers to quantify the efficiency of hydrolysis at least once in their studies.

### LC–MS/MS analysis

The hydrolyzed RNA samples were analyzed by LC–MS/MS using the protocols detailed in the following sections. In all cases, ribonucleosides were identified by a combination of retention time and fragmentation patterns confirmed with synthetic standards. To allow correction for inter-lab and day-to-day fluctuations in MS performance, all samples were spiked with 5-BrC as an internal standard.

### LC–MS/MS analysis at JGU

After digestion, all samples were spiked with 5-BrC and an assortment of [^13^C] isotopically labeled internal standards (SILIS) obtained from yeast.[30] A mixture of 500 ng of digested RNA, 50 ng of SILIS, and 250 pg of 5-BrC was injected, in three technical replicates, onto a Synergi 4 μm Fusion-RP 80 Å column (250 × 2.0 mm i.d.; Phenomenex, Aschaffenburg, Germany) maintained at 35°C. The column was eluted at 0.35 mL/min with 5 mM NH_4_OAc in water (pH 5.3) as solvent A and pure acetonitrile (ACN) as solvent B. The run started with 100% solvent A and proceeded through a linear gradient to 8% solvent B at 10 min and 40% solvent B after 20 min, before column regeneration at 100% solvent A for 10 min. The DAD was operated at 254 nm to acquire signals for the canonical ribonucleosides. The electrospray ionization (ESI) source was operated in positive-ion mode with optimized parameters as follows: drying gas temperature, 350°C; gas flow, 8 L/min; nebulizer pressure, 50 psi; sheath gas temperature, 350°C; sheath gas flow, 12 L/min; capillary voltage, 3000 V; and nozzle voltage, 0 V. The MS was operated in dynamic multiple reaction monitoring (dMRM) mode and controlled using Agilent MassHunter Workstation LC/MS Data Acquisition for 6400 Series Triple Quadrupole v. 10.1. Details of mass-to-charge ratios of precursor ions and product ions, and collision energies (CE) are included in Supplementary Table S2. Chromatograms were processed using Agilent MassHunter Workstation Qualitative Analysis v. 10.0. and raw peak areas for ribonucleosides were integrated using Agilent MassHunter Workstation Quantitative Analysis for QQQ v. 10.1

### LC–MS/MS analysis at MIT

The digested samples were centrifuged at 3000 x *g* at 4°C for 10 min before LC–MS/MS analysis. In each of the three technical replicates, 200 ng of digested RNA and 250 pg of 5-BrC were injected onto an Acquity BEH C18 column (50 × 2.1 mm i.d., 1.7 μm; Waters Corporation, Milford, MA, USA) operated at 25°C with a flow rate of 0.3 mL/min using mobile phase solvents Buffer A (0.02% formic acid in water) and Buffer B (0.02% formic acid in 70% aqueous ACN). The gradient of buffer B was as follows: 0–5 min, 0–1%; 5–7 min, 1–3%; 7–9 min, 3–7%; 9–10 min, 7–10%; 10–12 min, 10–12%; 12–13 min, 12–15%; 13–15 min, 15–20%; 15–16 min, 20–75%; 16–17 min, 75–100%; 17–20 min, kept at 100%, 20–21 min, 100–0%; and 21–25 min, kept at 0%. The DAD was operated at both 254 and 260 nm to monitor signals of canonical ribonucleosides. The JetStream ESI source was operated in positive-ion mode and optimized parameters as follows: drying gas temperature, 200°C; gas flow, 11 L/min; nebulizer, 20 psi; sheath gas temperature, 300°C; sheath gas flow, 12 L/min; capillary voltage, 3000 V; and nozzle voltage, 0 V. The MS was operated in dMRM mode and controlled using an Agilent MassHunter Workstation LC/MS Data Acquisition for 6400 Series Triple Quadrupole v. 10.1. CE was previously optimized for maximal sensitivity for each modification [31]. Retention times of modified ribonucleosides were confirmed with synthetic standards. Details of mass-to-charge ratios of precursor ions and product ions, and CE are included in Supplementary Table S2. Raw peak areas were extracted with Agilent Qualitative Analysis Navigator, Qualitative Analysis Workflows, and QQQ Quantitative Analysis (version B.08.00). Normalized signal intensities for each modified ribonucleoside were calculated by dividing each corresponding peak area by the peak area for the sum of the UV signals for the four canonical ribonucleosides.

### LC–MS/MS analysis at UC

Five microliters of the reconstituted samples containing 1 μg of digested RNA and 500 pg of 5-BrC were injected onto an Acquity HSS T3 column (1 × 100 mm i.d., 1.8 μm; Waters Corporation, Milford, MA, USA). The column was operated at 30°C with a flow rate of 0.1 mL/min using 5.3 mM NH_4_OAc in water (pH 4.5) as mobile phase A (MPA) and 40% ACN with 5.3 mM NH_4_OAc as mobile phase B (MPB). The gradient of MPB was as follows: 0% from 0 to 7.6 min, 2% at 15.7 min, 3% at 19.2 min, 5% at 25.7 min, 25% at 29.5 min, 50% at 32.3 min, 75% at 36.4 min to 36.6 min, 99% from 39.6 min to 46.8 min, and returning to 0% at 46.9 min. ESI parameters were as follows: static spray voltage, 3500 V; sheath gas pressure, 45 arbitrary unit; auxiliary gas pressure, 10 arbitrary unit; ion transfer tube value, 275°C; and vaporizer temperature, 250°C. The MS was operated in positive ion mode using selected reaction monitoring (SRM) mode. For all SRM transitions, collision gas (nitrogen) was set to 1.5 mTorr, dwell time was set to 150 ms, and chromatographic peak width was 55 s. Details of mass-to-charge ratios of precursor ions and product ions, collision energies, and RF values are included in Supplementary Table S2. Thermo Scientific Xcaliber software (Version 4.2.28.14) was used for data acquisition and processing.

### Absolute quantification of RNA modifications

Absolute quantification experiments were performed by JGU, MIT, and UC using all three laboratories’ protocols. Two aqueous stock solutions, Calmix A and Calmix B, containing a defined set of modified ribonucleosides (100 μM each) were prepared by JGU, aliquoted, and shipped to MIT and UC on dry ice (Supplementary Table S3A). Using their own 5-BrC solution as the internal standard for accounting for day-to-day variations in MS performance, the three laboratories prepared their calibration standards (0.1–500 nM) using an identical dilution protocol (Supplementary Table S3B). Calibration standards of canonical ribonucleosides for DAD (JGU and MIT) and LC–MS/MS (UC) analyses were also made independently in all three laboratories. At JGU and UC, each canonical ribonucleoside was accurately weighed and dissolved in water to prepare a stock solution. At JGU, stock solutions of the four canonical ribonucleosides were then mixed at a volume ratio of 1:1:1:1 and diluted to prepare calibration standards. At UC, stock solutions of the four canonical ribonucleosides were diluted individually to prepare calibration standards. At MIT, each canonical ribonucleoside was weighed and dissolved in solvents recommended by the manufacturers for absorbance measurements using a UV-Vis spectrophotometer. Briefly, A, G, and U were dissolved in 10 mM phosphate buffer (pH 7.0) and measured at 260, 280, and 262 nm, respectively, whereas C was dissolved in 0.1 M HCl and measured at 280 nm. Further dilutions were made until absorbance between 0.3 and 0.6 was achieved. Concentrations of the primary stock solutions were calculated using the Beer-Lambert law and the molar extinction coefficients listed in the certificates of analysis provided by the manufacturers (14.8, 13.2, 10.1, and 13.2 mM^−1^cm^−1^ for A, G, U, and C, respectively. A secondary stock solution of 400 μM was made by diluting the primary stock solution with RNase-free water. The four secondary stock solutions were then mixed at a volume ratio of 1:1:1:1: and serially diluted with RNase-free water to prepare calibration standards ranged from 2 to 100 μM. A calibration curve for each ribonucleoside was established by plotting its peak area against the number of moles injected onto the analytical column. Linear regression data and equations for calibration curves of ribonucleosides are listed in Supplementary Table S3C.

### Graphics and statistical analysis

Graphs were prepared and statistical analysis was performed using GraphPad Prism 10 software (GraphPad Software, Inc., San Diego, CA, USA), unless noted otherwise. RNA electropherogram data was exported from Agilent Bioanalyzer and TapeStation systems for comparative analysis. Statistical analysis of the effects of shipping conditions on RNA integrity was performed using an unpaired Student’s t-test. Correlations of RNA modification signals among different protocols and operators were evaluated using a Spearman correlation analysis. Statistical comparisons of the rank order of RNA modification levels among the labs were performed using a Kendall's coefficient of concordance (Kendall's W) in SPSS Statistics 26.0 (IBM, Armonk, NY, USA).

## Results

### RNA isolation

**Figure 2.**
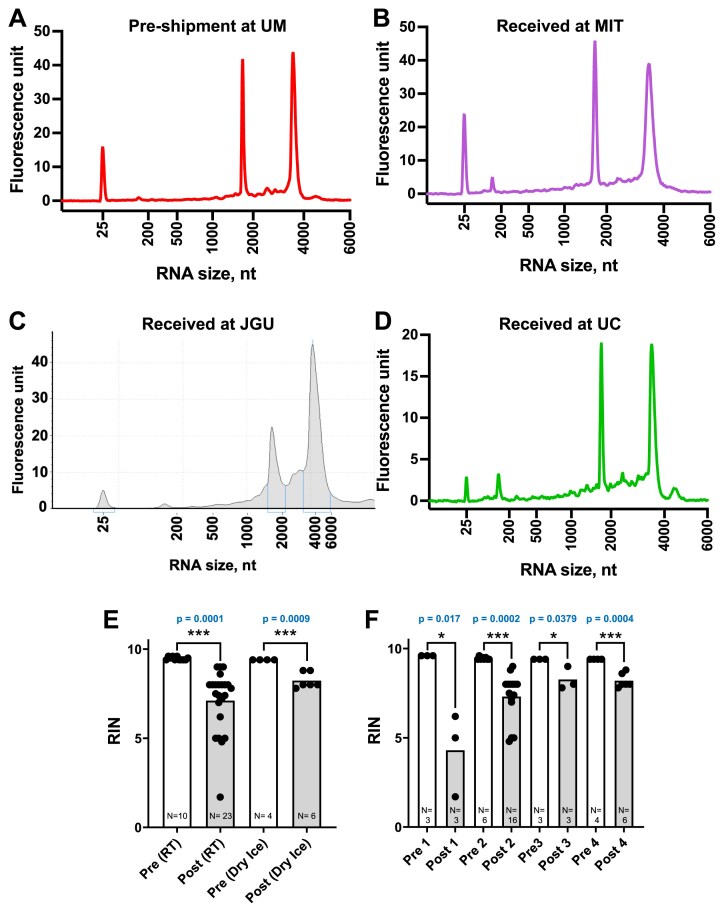
Assessment of RNA degradation during shipment. (**A–D**) Reconstructed RNA electropherograms for HEK293 total RNA samples prepared at UM (**A**) and following dry ice shipment in water to MIT (**B**), JGU (**C**), and UC (**D**). (**E**and**F**) Sample quality pre- and post-shipment based on RNA Integrity Number (RIN). Pre-shipment data from UM, post-shipment data from JGU, MIT, and UC. (**E**) Samples shipped at room (ambient) temperature (RT) or on dry ice. RT shipment resulted in significant RIN variation. (**F**) Samples were prepared under four different conditions (1–4 in Supplementary Table S4) and all were shipped on dry ice. Solution 4 (DEPC-treated water) best preserved RNA quality. Bar height in **E** and **F** represents mean with all data points shown (number of samples shown for each analysis) and statistical significance determined by an unpaired Student’s t-test.

HEK293 cells were chosen as the source of RNA for these studies based on the extensive study of this cell line for modifications of all major classes of RNA [10, 32, 33]. Using a commercial kit and RNase-free equipment and solutions [34], total RNA was isolated from HEK293 cells at UM as the common RNA standard for LC–MS/MS analysis at JGU, MIT, and UC. Quality control analysis using an Agilent Bioanalyzer showed clearly defined peaks for 18S and 28S ribosomal RNAs (Fig. 2A, Supplementary Fig. S1), with consistently high RIN values of 9.58 ± 0.08 (*n* = 9) for repeated isolations (Supplementary Fig. S1). The purified RNA was stored at -80°C before and after shipping, with no evidence of significant degradation as judged by Bioanalyzer and TapeStation analyses (Fig. 2). To ensure maximum consistency, all data presented hereafter on shipping and LC–MS/MS analysis were generated with aliquots of a single RNA preparation.

It is important to note that all equipment and solutions used for RNA purification, processing, and shipment should be free from ribonuclease (RNase) contamination. Among the methods for preparing RNase-free water to use for all solutions, such as simple autoclaving, ultrafiltration, and treatment with RNase-inhibitors [34–36], we chose to use diethylpyrocarbonate (DEPC) treatment followed by autoclaving to remove residual DEPC [34, 36]. This choice was validated by the high RNA quality RIN numbers achieved in our studies (Fig. 2).

### RNA preparation and shipping

We next evaluated methods to prepare RNA samples for shipment to three laboratories for LC–MS/MS analysis, focusing on preserving RNA integrity. Following elution from Qiagen columns, RNA samples were processed

according to the four shipping conditions detailed in Supplementary Table S4. RIN values before shipment and after arrival at each laboratory are detailed in Fig. 2E and F. Comparing the results between shipment at ambient temperature (RT) and on dry ice (Fig. 2E), RIN values decreased significantly during shipment regardless of temperature; dry ice was still present in the shipping boxes on arrival in the three labs. Degradation was less pronounced for samples shipped on dry ice (an average decrease of 1.2 RIN) compared to those shipped at RT (an average decrease of 2.4 RIN). Moreover, samples shipped on dry ice exhibited more consistent RIN values, while those shipped at RT were highly variable, possibly due to uncontrollable shipping conditions. Based on these findings, we conclude that RNA samples should be shipped on dry ice to minimize degradation.

We next assessed the effects of four conditions for preparing the RNA for shipping (Supplementary Table S4); in all cases, samples were then shipped on dry ice. As shown in Fig. 2F, samples prepared in 0.5 M NH_4_OAc with three volumes of EtOH showed high variability in RIN values, making this condition unsuitable for further use. Of the remaining three conditions, RNA shipped in DEPC-treated water showed the least degradation across all three laboratories (Fig. 2F). It required minimal sample manipulation (elution of RNA from the purification column with DEPC-treated water), which likely contributed to the observed stability. Based on these results, we conclude that shipping RNA in DEPC-treated water on dry ice is the optimal method to preserve RNA integrity for downstream analyses. This finding is crucial since commercial shipping between continents regularly encounters issues regarding import clearance for customs, biohazards, and transportation safety. Because of such delays in about half of the shipments from the USA to Germany, the importance of the following cannot be overstated: (1) shipping the RNA samples with enough dry ice to last at least 4–5 days, (2) ensuring that all required customs paperwork is provided to the shipping company, (3) keeping backup aliquots of RNA in the outgoing laboratory for re-shipment in case the sample degrades, and (3) performing RNA quality checks before and after shipment.

### Protocols for LC–MS/MS analysis of RNA modifications

Using the high-quality RNA shipped from UM, we proceeded to compare the analysis of RNA modifications by LC–MS/MS in laboratories at JGU, MIT, and UC. In preparation for this analysis, it is important to identify the critical steps in the LC–MS/MS analytical method: (1) identification of the specific modified ribonucleosides being targeted for analysis, (2) conditions for hydrolysis of the RNA to ribonucleosides, (3) the HPLC elution conditions for resolving the ribonucleosides from one another, (4) the MS settings to optimally detect and quantify the ribonucleosides, and (5) the approach to normalizing MS signal values to account for varying amounts of injected RNA and for day-to-day and machine-to-machine variance in ribonucleoside signals. It is important to define our use of the term “quantification” for both relative and absolute quantification of RNA modifications. An analytical method is considered quantitative if the signal is proportional to concentration of the analyte and if the signal is measured in the linear range of the method [37]. Both requirements are met for the studies presented here. Further, the quality of quantitative methods is measured in terms of precision (i.e. the variance in repeat measurements of the same sample) and accuracy (i.e. how close the measurement is to the true value) [37]. While relative quantification can only be precise and absolute quantification can be both precise and accurate, we consider both to be quantitative.

The first step is to define a list of modifications that could plausibly be expected in total RNA from human cells. We curated a list of known human modifications [38, 39] and added ribonucleosides from lower eukaryotes and several prokaryote-specific modifications as negative controls to test the specificity of our approach and to monitor potential contaminations during cell culture, RNA isolation, and work-up. The list of 56 modifications is detailed in Supplementary Table S2 with their Modomics code [40].

**Figure 3.**
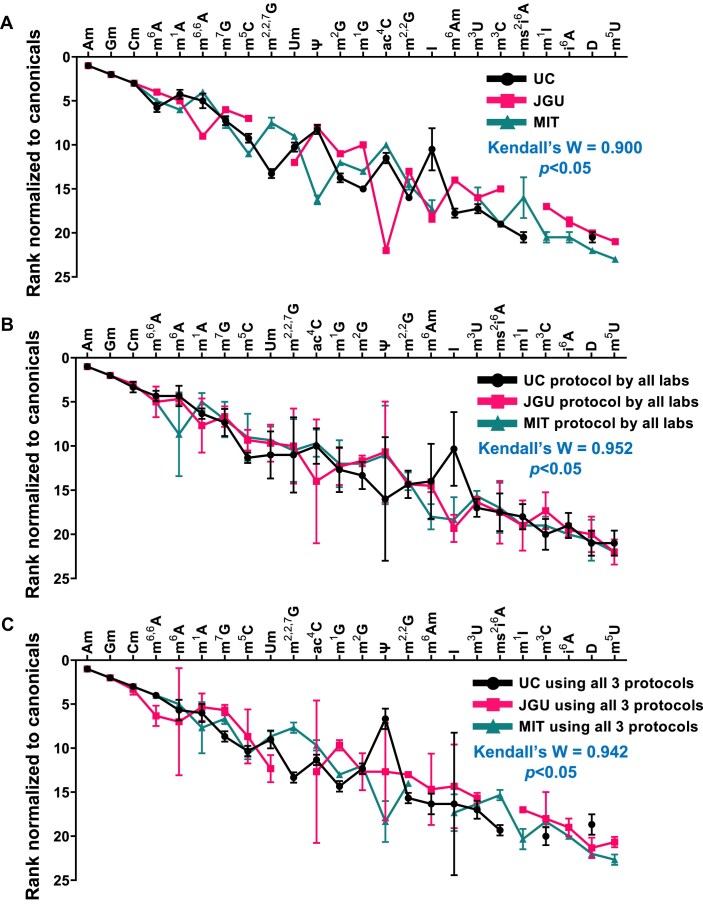
Interlaboratory comparisons of LC–MS/MS normalized signal intensities for 24 RNA modifications. Data are presented as the rank order of signal intensity normalized to canonical nucleosides (1 for highest signal, 24 for lowest signal). In all cases, each lab analyzed four different RNA samples in three technical replicates, depending upon how protocols were being compared. The triplicate data were averaged and data are presented as mean ± SD for N = 4 RNA samples. Data used to prepare these graphs are presented in Supplementary Table S5 and representative extracted ion chromatograms are shown in Supplementary Fig. S2. (**A**) Variance across the three labs: each lab performed triplicate analyses of the RNA using the lab’s RNA hydrolysis protocol. The rank order of normalized signal intensities was then determined for each lab and plotted as shown. (**B**) Variance due to the protocol: each lab analyzed the RNA samples (N = 4) using each of the three lab-specific RNA hydrolysis protocols. For each protocol, the lab-specific values were averaged to determine the rank order. (**C**) Variance due to the operator (i.e. lab): each lab analyzed the RNA using each of the three lab-specific RNA hydrolysis protocols (triplicate analysis for each protocol for four samples), calculated the rank order for each protocol, and then calculated the mean ± SD across the three protocols. (**A-C**) Interlaboratory agreement was assessed using Kendall’s coefficient of concordance (**W**), where 0 denotes “no agreement” and 1 denotes “complete agreement”. Modified ribonucleosides that were not detected in all three datasets were excluded in Kendall's W analysis. Significant agreement was observed when *P* < 0.05.

The second step involves RNA hydrolysis. All three labs shared the same fundamental features of nucleases to degrade the RNA to ribonucleotide level and a phosphatase to convert ribonucleotides to ribonucleosides for optimal HPLC resolution. Here we are considering the impact of lab-specific variations in the digestion protocol on the quantitative analysis. For example, each team used different enzyme mixtures: JGU used benzonase, nuclease P1, bovine intestinal alkaline phosphatase, and snake venom phosphodiesterase; MIT used benzonase, calf intestinal alkaline phosphatase, and snake venom phosphodiesterase; and UC used nuclease P1, FastAP thermosensitive alkaline phosphatase, and snake venom phosphodiesterase. JGU and MIT added adenosine deaminase inhibitors pentostatin or coformycin, respectively, which prevents adventitious adenosine deamination to inosine during RNA hydrolysis. MIT also used antioxidants BHT and DFOA to prevent oxidative damage and oxidation of ho^5^U and ho^5^C during hydrolysis. While we did not quantify the efficiency of the RNA hydrolysis in the present experiments by comparing Bioanalyzer or HPLC profiles before and after hydrolysis, previous assessments of the efficiency of RNA hydrolysis over the past two-decades [12, 21, 41] suggest that the RNA is fully hydrolyzed ribonucleosides. We compensated for potential hydrolysis bottlenecks by using incubation times significantly longer than required based on experience and vendor specifications. Attempts to calculate the hydrolysis efficiency by summing the quantities of the canonical ribonucleosides as total output RNA and comparing this value to the total input RNA calculated from the Nanodrop or other estimated RNA concentration yielded hydrolysis efficiencies ranging from 63% to 118% (Supplementary Table S3). However, we are suspect of this calculation due to significant errors in quantifying RNA by Nanodrop or other spectroscopic methods. The important point here is that, while RNA hydrolysis conditions (see Materials and Methods) did lead to variations in the detected levels of some modifications that we note below, all three labs found similar and consistent levels canonical nucleosides in all samples based on normalization data and absolute quantification data (Supplementary Tables S3 and S5), and there was very strong agreement in the quantitative analysis of ribonucleosides among the labs (Fig. 3) as discussed shortly. Of course, readers are advised to determine the efficiency of hydrolysis for their experimental conditions.

The third and fourth steps of HPLC conditions for resolving ribonucleosides and the MS settings for detecting and quantifying them also varied among the three labs. All labs used C18 reversed-phase HPLC columns to resolve the ribonucleoside mixtures, but variations in solid phase properties, column dimensions, solvent composition, and flow rates led to different HPLC retention times for the target ribonucleosides, as detailed in Supplementary Table S2. Similarly, though all three labs used the same fragmentation transitions for each ribonucleoside (Supplementary Table S2), the three different MS systems resulted in variations in the optimal CE needed for each ribonucleoside (Supplementary Table S2). Again, these differences could contribute to variations in the detected levels of modified ribonucleosides.

Finally, the fifth step of data processing involved identifying a common signal normalization method across the three analytical laboratories for relative quantification of ribonucleosides and preparing calibration curves for signal interpolation for absolute quantification of ribonucleosides. To account for variations in the amount of input RNA represented in the hydrolysate injected on the HPLC column, the machine-generated signals for each ribonucleoside are divided by a metric for the amount of one or all canonical ribonucleosides also present in the sample. The canonical ribonucleosides can be quantified in two ways. Two labs (JGU, MIT) used an inline UV detector positioned between the analytical column and the MS to record the UV absorbance for the canonical ribonucleosides and then sum the four absorbance values to normalize the signals of modified ribonucleosides. UC used the MS signals for the canonical ribonucleosides to perform the normalization. In this case, care must be taken to ensure that the signal for each canonical ribonucleoside is within the linear range of the MS response, which is challenging since abundances of the canonicals are orders-of-magnitude larger than the modified ribonucleosides. Similarly, to account for day-to-day variation in MS performance, all three labs added a known amount of 5-BrC to each sample. For absolute quantification, JGU provided two mixtures of ribonucleoside standards (Supplementary Table S3A) for the three labs to use in preparing calibration curves to interpolate MS signals for the ribonucleosides (Supplementary Table S3B,C). The calibration curves allow conversion of the ribonucleoside machine signal to moles or mass, which are then normalized to the absolute quantity of the canonical nucleosides.

### Analysis of modified ribonucleosides in total RNA by three labs

Based on the protocols detailed above, laboratories at JGU, MIT, and UC performed LC–MS/MS analysis of the modified ribonucleosides detectable in high-quality total RNA isolated from HEK293 cells and shipped using the optimized shipping conditions by UM. All three labs detected 17 modifications in this initial run, with 7 more detected by at least two labs. The 24 modified ribonucleosides detected in total are shown in Fig. 3 and a representative extracted ion chromatogram is shown in Supplementary Fig. S2A. As expected, most of the detected modifications were simple methylations on the base and ribose moiety, along with other simple modifications like pseudouridine (Ψ), dihydrouridine (D), I, and *N*^4^-acetylcytidine (ac^4^C). This set of modified ribonucleosides was not surprising given the proportions of heavily modified tRNAs and moderately modified rRNAs in the total RNA sample analyzed [10–12, 16]. Also unsurprisingly, there were differences among the laboratories in detecting specific modifications. Since direct comparisons of normalized MS signal intensities are not reasonable due to instrumental differences, we quantified the rank order of the normalized MS signal intensities for the 24 modifications across the three laboratories (Fig. 3A; 1 for highest signal, 24 for lowest signal). This analysis revealed a significant consistency with Kendall's coefficient of concordance (W) of 0.900 and *P*< 0.05. The concordance was the strongest for the most abundant modifications, such as 2′-*O*-methyladenosine (Am), 2′-*O*-methylguanosine (Gm), and 2′-*O*-methylcytidine (Cm). The modifications showing the highest variation among the laboratories were ac^4^C, Ψ, and I. ac^4^C is hydrolytically unstable, [42] so differences in the pH or processing time for RNA isolation and hydrolysis could account for the variance in detecting this modification. Conversion from A to I is well established to be caused by contaminating deaminases in commercial enzyme preparations [21, 43]. To suppress this artifact, two labs (JGU, MIT) added adenosine deaminase inhibitors, coformycin or pentostatin, during RNA processing [21, 43]. The basis for variable detection of Ψ is not known but could result from variations in the efficiency of RNA hydrolysis at sites containing Ψ or from machine-specific differences in fragmentation of this C-glycosidic ribonucleoside [44].

We next performed two sets of studies to better understand the factors that determined the variance in detecting the RNA modifications across the three labs. To assess variance due to the protocol, each lab performed triplicate analyses of the RNA using each of the three lab-specific RNA hydrolysis protocols and, for each protocol, calculated the rank order as in Fig. 3A. As shown in Fig. 3B, the concordance was slightly stronger for this assessment of protocols than for interlab variance (W = 0.952 versus W = 0.900), with the MIT and JGU protocols showing very strong consistency compared to the UC protocol. Again, the widest variations were observed for Ψ and I. A second set of studies assessed the contribution of the operator (i.e. the lab) to variance, which reflects the LC–MS/MS system and other lab-specific factors. Here, each lab performed three technical replicate analyses of the RNA samples using each of the three lab-specific RNA hydrolysis protocols. Each lab then calculated the rank order for each protocol and then calculated the mean ± std dev for the rank order values across the three protocols. The plot in Fig. 3C shows that there is again strong concordance among the three labs, with the widest variation observed for Ψ. It is not clear that there is any significant difference overall between the operator and the protocol in the quantification of modified ribonucleosides by LC–MS/MS (W = 0.942 versus W = 0.952, respectively; Fig. 3B and C).

**Figure 4.**
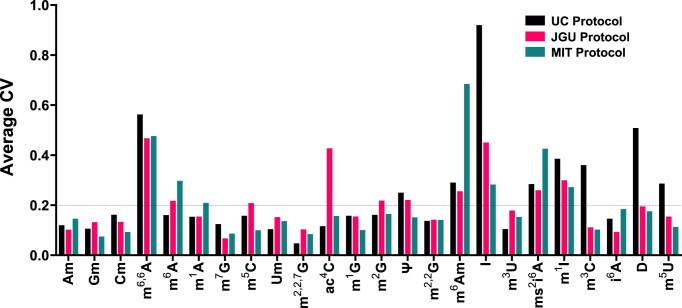
Protocol-specific coefficients of variation (CV) for 24 modified ribonucleosides. The CV for the normalized signal intensity for each ribonucleoside was calculated from the three replicate analyses performed by each lab with each protocol. The average CV for each protocol across the three laboratories was then determined and plotted for each protocol. The dotted line at CV = 0.2 indicates strong precision. Data used to prepare this graph are presented in Supplementary Table S5.

To obtain a more granular view of the variance in quantifying each of the 24 ribonucleosides, we determined the coefficient of variation (CV) for the normalized signal intensity for each ribonucleoside in the three replicate analyses performed by each lab with each protocol. Based on the CV results from each lab (Supplementary Fig. S3), we calculated the average CV for each protocol across the three laboratories. As shown in Fig. 4, the CVs for measurements made with the three protocols were similar for most modifications, with 19 of 24 modifications showing a CV of less than 0.2 for at least two of the three labs. There is thus reasonably strong analytical precision across the labs [45]. CVs for several modifications (ac^4^C, D, I, m^3^C, m^6^Am, and ms^2^i^6^A) were consistently high no matter which protocol was used for the analysis (Fig. 4).

**Figure 5.**
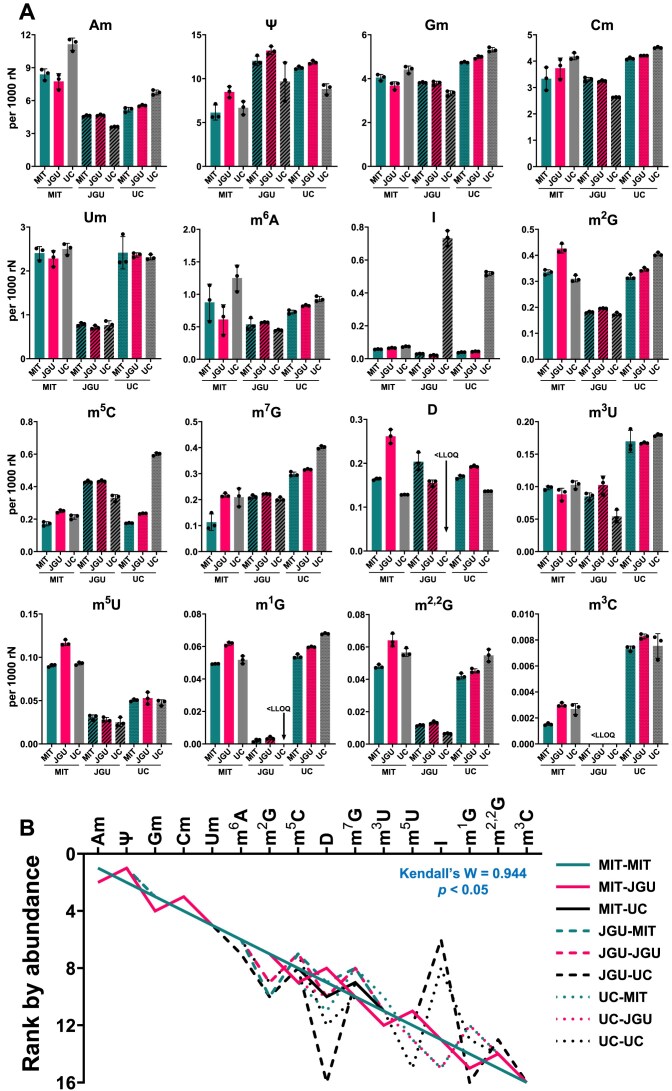
Interlaboratory comparison of absolute quantification of 16 RNA modifications by LC–MS/MS. Absolute quantification was performed by three labs using a common set of calibration standards (Supplementary Table S3). (**A**) MIT, JGU, and UC each performed absolute quantification of 16 ribonucleosides using each of the three hydrolysis protocols (labels under each bar). Data for individual ribonucleosides are presented as box and whisker plots showing the mean, standard deviation, and individual data points. (**B**) Rank order plot for the analyses by three labs. Interlaboratory agreement was assessed using Kendall's coefficient of concordance (**W**), where 0 denotes “no agreement” and 1 denotes “complete agreement”. Significant agreement was observed when *P* < 0.05. Data used to prepare these graphs is presented in Supplementary Table S5 and representative extracted ion chromatograms are shown in Supplementary Fig. S2. <LLOQ, less than the lower limit of quantification.

**Figure 6.**
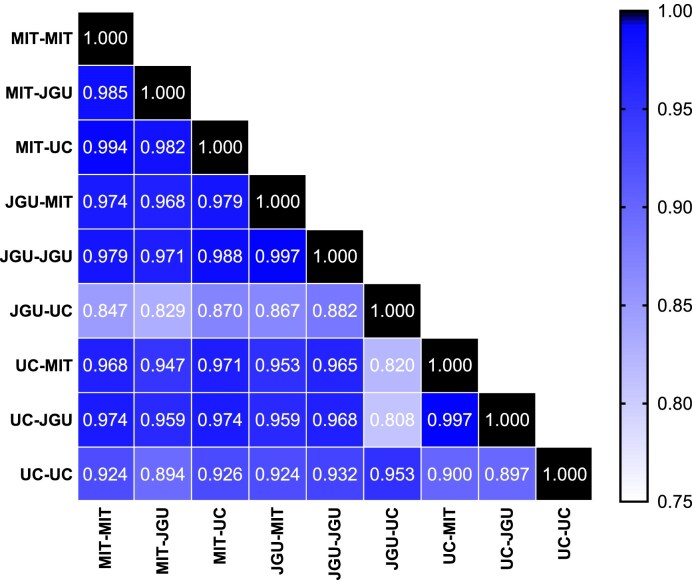
Spearman correlation analysis of absolute quantities of 16 RNA modifications determined by three labs using each of the labs’ protocols for absolute quantification. “MIT-MIT” denotes MIT performing the MIT protocol, “MIT-JGU” denotes MIT performing the JGU protocol, and so on. Data are presented as Spearman correlation coefficients, with 0.8–1.0 indicating very strong monotonic correlation and significance at *P* < 0.05. Data used to prepare these graphs are presented in Supplementary Table S5.

Finally, relative quantification with normalized signal intensities can provide precise values for changed modification levels between samples but lacks information about the true level of the modified ribonucleosides that is provided by absolute quantification. Here it is important to point out that absolute quantification can be achieved by any of several approaches, the rigor of which differs in terms of the accuracy of quantification. Using a single universal internal standard provides the least accurate signal calibration for the wide range of ribonucleoside structures with variations in charge and polarity. While calibration with isotope-labeled internal standards ensures the most accurate quantification [30], it is costly and impractical for most researchers. We performed external calibration with unlabeled standards, which is nearly as accurate, significantly less expensive, and more widely available to the research community. We do not recommend using 5-BrC as an internal standard for calibrating ribonucleoside signals and recommend instead using this standard, as intended for quality control, to correct for day-to-day variations in machine performance and thus improve the precision of separate analyses. Here, all three labs repeated the LC–MS/MS analyses of total RNA using known amounts of synthetic standards to prepare calibration curves to convert the ribonucleoside machine signals to moles of ribonucleoside. The three labs shared the same chemical standard mixture of the ribonucleosides to further pinpoint the source of differences between laboratories, since the use of a common chemical standard compensates for lab-specific differences such as varying detection efficiencies for different nucleosides. Calibration curves for ribonucleosides were prepared as described in the Materials and Methods and good linearities were obtained with all coefficients of determination (R^2^) exceeded 0.96 (Supplementary Table S3C). Among the 27 ribonucleosides present in the common standard mixture, 16 were detected and quantified as the number of modifications per 1000 canonical ribonucleosides (Fig. 5); a representative extracted ion chromatogram is shown in Supplementary Fig. S2B. Overall, the ranking of the quantitative results is significantly different from the signal-intensity-based ranking seen in Fig. 3, illustrating the fact that the machine signals do not correlate well with the true abundance of the modified ribonucleosides due to different ionization efficiencies in the ESI source among other factors. This is especially true for Ψ, which is the most abundant modification with ∼1% of canonical ribonucleosides and similarly for Um. Care must be taken in interpreting the data in Fig. 5. While 7 modified ribonucleosides consistently show greater than 2-fold difference levels among the three labs (e.g. Am, Um, m^2^G, m^5^C, m^2,2^G, m^5^U, and m^3^C), there is a 10 000-fold range in ribonucleoside levels from 1 per 10^6^ nt to 1 per 10^2^ nt (Fig. 5). This large range diminishes the impact of the 2-fold differences for some modifications. While there were modifications showing laboratory-specific over- or under-reporting (e.g. m^5^U, Ψ, Um), there were also some protocol-specific differences such as the JGU and UC analyses performed with the UC protocol lacking deaminase inhibitor, where A-to-I deamination led to a ∼ 5- to 7-fold increase in I. The strong concordance of the absolute quantification data for the three labs is shown in the Spearman correlation analysis presented in Fig. 6. This analysis revealed Spearman correlation coefficients r > 0.80 with *P*-values ≤ 0.0003, indicating significantly strong correlations among the three datasets. We also calculated the CVs for the analyses performed by each lab (Supplementary Fig. S4; Supplementary Data Table S5), which again shows that no one protocol performs markedly better than the others. Importantly, however, the overall precision of the analyses improved by roughly two-fold, with a CV of approximately 10% now apparent for all three labs using all three protocols (Supplementary Fig. S4). The improvement of CV with calibration is a well-established phenomenon [46].

## Discussion

Given the increasing focus on the epitranscriptome in health and disease, we sought to evaluate the robustness of all steps involved in LC–MS/MS analysis of RNA modifications and identify optimal protocols. The analysis of modified ribonucleosides by LC–MS/MS is one of several tools available to researchers for studying the epitranscriptome and it is most useful for defining the spectrum of modifications in an organism [47], for quality control in the synthesis of modified RNAs (e.g. 5′-caps, m^1^Y content [18, 48]), and for rigorous quantitative analysis of changes in the modification levels in cells under different conditions [47, 49, 50]. While this approach of RNA hydrolysis into ribonucleosides excels at quantifying modification levels and systems-level modification analysis, it sacrifices information about the position of modifications within RNA sequences. LC-MS quantification of individual ribonucleosides in RNA is thus complementary to next-generation and Nanopore-based sequencing and to the emerging application of LC-MS to RNA modification mapping [6, 8, 10]. The goal of this effort is to provide the research community with a comparative evaluation of protocols that can be readily adopted by individual laboratories or core facilities, enabling the processing of RNA samples for modification analysis and facilitating precise, accurate, and globally comparable epitranscriptome data. The comparison of protocols among the three analytical labs demonstrated both the strengths of existing methods for RNA modification analysis and the need for optimization of the various steps. The initial analyses achieved a strong concordance for both relative and absolute quantification and revealed the need for artifact control and establishing the optimal parameters for and limits of detection of available mass spectrometry systems.

Surprisingly, the technically simplest step of shipping RNA samples to the analytical labs proved to be one of the largest sources of error (Fig. 2). This is a critical step since most researchers will not have access to mass spectrometry facilities or the expertise to perform LC–MS/MS analyses and must send samples to collaborators or transport the RNA to a core facility for analysis. The importance of maintaining the integrity of purified RNA cannot be overstated given the risk of biased loss of specific RNA molecules and the contamination of small RNAs with fragments of larger RNA (i.e. low RIN values; Fig. 2A–D). We determined that the only reliable method for long-distance transport of RNA with minimal degradation was to prepare the purified RNA in RNase-free water (e.g. DEPC-treated) and send the sample on dry ice (Fig. 2E and F). We also found that storing RNA in a buffered solution (e.g. NH_4_OAc) was significantly less effective than using nuclease-free or DEPC-treated water, suggesting that certain buffer components may introduce trace contaminants that compromise RNA stability. Given its accessibility and effectiveness in preserving RNA integrity, self-prepared or commercial nuclease-free water is also recommended for both short- and long-term RNA storage and shipping.

The protocol for hydrolysis of RNA to ribonucleosides introduced significant contributions to variability despite the overall concordance of the quantitative analyses (Fig. 3). Major risks introduced by RNA hydrolysis include degradation of unstable modifications such as ac^4^C and ct^6^A and enzymatic deamination of A to I and C to U in modified ribonucleosides containing these nucleobases [15, 43]. In the absence of an adenosine deaminase inhibitor such as pentostatin or coformycin, we observed artificially high levels of inosine, which can be largely suppressed with the inclusion the inhibitors (Fig. 3). While JGU used pentostatin and MIT used coformycin at concentrations 10^2^- to 10^6^-times their adenosine deaminase binding constants [51] in the hydrolysis reaction, there was still significant variability in Inosine levels (Fig. 4). This suggests other sources of adenosine deaminase contamination (e.g. cell lysis, RNA purification). Note that coformycin is no longer commercially available. The need to use tetrahydrouridine (THU) to inhibit cytidine deaminases is less clear. We have not observed any differences in the presence or absence of THU in nearly two decades of DNA and RNA studies, so we do not routinely add it to the hydrolysis mixtures [12, 21, 41]. One recent study noted inconsistent changes in the levels of m^5^C, Cm, and the m^5^U deamination product of m^5^C in the presence and absence of THU [15]. The reader should determine the best case for the use of THU in their analysis of specific modifications. For example, contamination of THU with dihydrouridine (D) is a potential concern for accurate measurement of D in tRNA, which warrants repurification of THU solutions by HPLC or other methods to remove ribonucleoside-like contaminants [15]. In any event, there is strong historical precedent for contamination of phosphatases and other enzymes with adenosine deaminase activity [12, 21, 41], so addition of pentostatin or coformycin is highly recommended to inhibit this activity. The consequences of omitting an adenosine deaminase inhibitor were clearly demonstrated in the present studies.

Chemically unstable modifications also contributed to the inter-laboratory variability. While neither the tRNA position 37 modification t^6^A nor its ring-closed cyclic counterpart ct^6^A were detected in the total RNA analyzed by any of the labs, likely due to dilution of their signals in total RNA, ct^6^A is an example of a modified ribonucleoside well known to be sensitive to ring opening at alkaline pH (stable in acidic pH) and adduct formation with primary amines (e.g. Tris buffer). By performing RNA digestion at neutral pH and shortening reaction time, Miyauchi *et al.* observed a significant increase in ct^6^A and a decrease in t^6^A [52], suggesting that a significant portion of what most analysts observe as t^6^A should actually be ct^6^A.

Similarly, ac^4^C is sensitive towards deacetylation at alkaline pH and in the presence of nucleophilic amines [20]. When using the JGU protocol, we found a greater variation in the ac^4^C levels (Fig. 3 and 4). We first attributed this to the hydrolysis step but specific effects of salts, pH, temperature, and incubation time do not explain the large variability experienced in the JGU lab (Fig. 4) since these factors are all constant among the experimental replicates. Other factors such as operator variation in the experimental schedule, pipetting errors, or changes in LC or MS performance (e.g. sensitivity, noise level) between replicate analyses must be considered. Such idiosyncrasies partly account for the variable detection of specific modifications by individual labs, such as m^2,^ [2]^, 7^G missing in some of the JGU analyses and m^6^Am missing in some of the MIT analyses in Figs 3A and C. Machine-specific issues such as sensitivity likely partly explain inter-laboratory variability. For example, the higher CVs for UC measurement of m^3^C, i^6^A, D, and m^5^U compared to MIT and JGU (Fig. 4) and the occasional absence of these modifications in the UC analyses (Fig. 3A and C) may be due to machine-specific sensitivity for these low-abundance modifications—the rank ordering positions the modifications with the lowest signals to the right side of the graphs in Fig. 3. This is consistent with the imprecision of measuring small signals near the limit of detection of the instrument or close to the noise level [45]. Sensitivity problems can often be overcome by loading more RNA onto the LC–MS/MS system without overloading the HPLC column or MS ionization and detection systems.

In addition to sensitivity, there are other operator- and machine-specific contributions to inter-laboratory variability, such as operator inconsistency in RNA isolation, purification, hydrolysis, injection on the HPLC column, and data analysis. Matrix effects, defined as the change in the measured signal of a ribonucleoside due to the presence of other components in the sample, can arise from residues left from RNA processing and purification and interfere with ionization efficiency of the ribonucleoside. The performance of HPLC columns and MS systems degrades over time, requiring column cleaning or replacement and MS cleaning, tuning, or parts replacement.

Despite these many potential contributors to variability among labs in detecting and quantifying modified ribonucleosides, the agreement among the three experienced labs involved in this study was remarkably high. Here it is important to note that the present studies represent the first attempt to define the analytical variability for LC-MS-based epitranscriptome analyses among different labs. Our experience here and in our publications [30, 42, 53] has revealed a CV of roughly 20%, which may represent a potential community standard for simultaneous determination of normalized signal intensities for all RNA modifications in a biological sample by LC-MS. When calibration is included, the CV improves to about 10%, which is consistent with the large body of analytical chemistry literature [46]. Future studies will be required to formally define the acceptable variability for ribonucleoside analyses in critical manufacturing and diagnostic applications. This is not a trivial undertaking since the CV for each ribonucleoside will be different even in the simultaneous analysis of all the modifications, due to factors such as abundance (i.e. higher CV for modifications closer to the limit of detection), chemical instability (i.e. m^1^A, ct^6^A, ac^4^C, *et al.* [42]), artifactual formation (e.g. I), and MS efficiencies of ionization, fragmentation, and detection.

One issue that we did not address here since we all used the same RNA sample prepared by UM is the lack of a standard form of modified RNA for researchers to use as a benchmark for validating their studies. While some groups have used total RNA from organisms such as yeast as a reference material for LC-MS-based RNA modification analysis, this approach has significant limitations. The abundance of specific RNA modifications varies widely depending on growth conditions, media composition, strain background, growth phase, and environmental stressors. These variables make total RNA from natural sources unreliable as a consistent benchmark across laboratories or experiments. Synthetic RNA oligonucleotides containing specific modifications at known positions, or mixtures of purified, quantified modified ribonucleosides, could provide a more reproducible and accurate approach for benchmarking LC-MS performance. However, this approach suffers from the unfeasibility of incorporating all ∼50 human modifications into oligonucleotides or accounting for sequence-selectivity of hydrolysis efficiency. The absence of such standards currently limits our ability to assess accuracy, reproducibility, and cross-platform comparability in RNA modification analysis. Addressing this gap is essential for advancing the rigor and utility of LC-MS-based methods in the field.

Here we make the following recommendations for minimizing errors and variability in the LC–MS/MS analysis of modified ribonucleosides:

- Perform quality control checks at each step of the process (cell lysis, RNA extraction, RNA purification, hydrolysis, HPLC resolution), at least for the first few analyses for experiential learning and always for defining the quality of each RNA sample. Do not use samples with RIN < 8.
- Ship RNA samples in DEPC- or nuclease-free water on dry ice, using enough dry ice to ensure days-long delays due to shipping or customs problems.
- The optimal hydrolysis protocol is one that uses any of the enzymatic mixtures and buffer systems described here by the three labs, with addition of (1) A and C deaminase inhibitors and (2) antioxidants. Special attention to unstable modifications requires unique conditions for each one, including the buffers and pH of the cell lysis and RNA purification steps. There is no single optimal method for all 170 known modified ribonucleosides or the 50 + human RNA modifications: practitioners must optimize for modifications of interest. The three protocols presented here all work well.
- It is important to at least once assess the completeness of RNA hydrolysis using a Bioanalyzer or HPLC to analyze the reaction mixture for the presence of undigested RNA fragments, or, as we did here, to perform absolute quantification of canonical ribonucleosides to back calculate the efficiency of hydrolysis.
- Optimize the HPLC and MS parameters unique to your instruments and protocols, with optimization of MS parameters specific for each ribonucleoside to maximize sensitivity.
- For determination of normalized signal intensities, we recommend using an inline UV spectrophotometer to quantify canonical ribonucleosides for signal normalization. However, MS signals for the A, G, C, and U can be used if it is established that the signal intensity falls within the linear response range of the MS detector. A reasonable workaround is to perform two injections: one undiluted to capture the signals for the modified ribonucleosides and one diluted 10-fold to capture the signals for the canonical ribonucleosides.
- The choice of absolute versus relative quantification depends on the research question and resources available to the researcher. Absolute quantification with internal or external calibration improves both accuracy and precision but may not be practical in all situations. Careful attention should be given to rigorous validation of the concentrations of ribonucleoside solutions used for calibration, with UV absorbance determination of concentration at a minimum.
- In all cases and for all steps, optimal LC–MS/MS epitranscriptome analyses require constant attention to instrument maintenance and to the precision and accuracy of processing times, solution composition, and operating parameters.

The LC–MS/MS comparison presented here represents a critical step toward establishing universally applicable methods for RNA modification analysis, supporting laboratories worldwide in their efforts to interrogate the RNome with precision and reliability.

## Supplementary Material

gkaf895_Supplemental_Files

## Data Availability

Raw LC–MS/MS data have been deposited to the ProteomeXchange Consortium via the PRIDE (https://www.ebi.ac.uk/pride/) partner repository with the project accession #PXD061704. All other data are included in Supplementary files.
