# Supplementary material for: Toward standardized epitranscriptome analytics: an inter-laboratory comparison of mass spectrometric detection and quantification of modified ribonucleosides in human RNA
Source: Nucleic Acids Res. 2025 Sep 12;53(17):gkaf895. doi: 10.1093/nar/gkaf895 (PMC12445667; doi:10.1093/nar/gkaf895)
Supplement: gkaf895_Supplemental_Files [file gkaf895_supplemental_files.zip › Hengesbach et al Supplementary Information 2025 6-19.pdf]

Supplementary Information for
Toward standardized epitranscriptome analytics: An inter-laboratory comparison of mass spectrometric detection and quantification of modified ribonucleosides in human RNA

Martin Hengesbach, Chi-Kong Chan, Tulsi Bhandari, Alan Bruzel, Michael S. DeMott, Ganna Podoprygorina, Guangxin Sun, Ellen Tabeling, Vivian G. Cheung, Peter C. Dedon, Mark Helm, Patrick A. Limbach

Contents:

• **Supplementary Figure S1**: Agilent Bioanalyzer tracings for the 9 preparations of total RNA from HEK293 cells at UM

• **Supplementary Figure S2**: Representative total extracted ion chromatograms from relative and absolute quantification of ribonucleosides by LC-MS.

• **Supplementary Figure S3**: Coefficient of variation results for each lab performing all three protocols.

• **Supplementary Table S1**: Sources of materials and reagents

• **Supplementary Table S2**: RNA modifications included in the analysis. See separate Excel spreadsheet.

• **Supplementary Table S3**: External calibration standard stock solutions, working solutions, and linear regression data for absolute quantification experiments. See separate Excel spreadsheet.

• **Supplementary Table S4**: Four conditions for preparing RNA for shipping

• **Supplementary Table S5**: Data for Figures 3-5. See separate Excel spreadsheet.

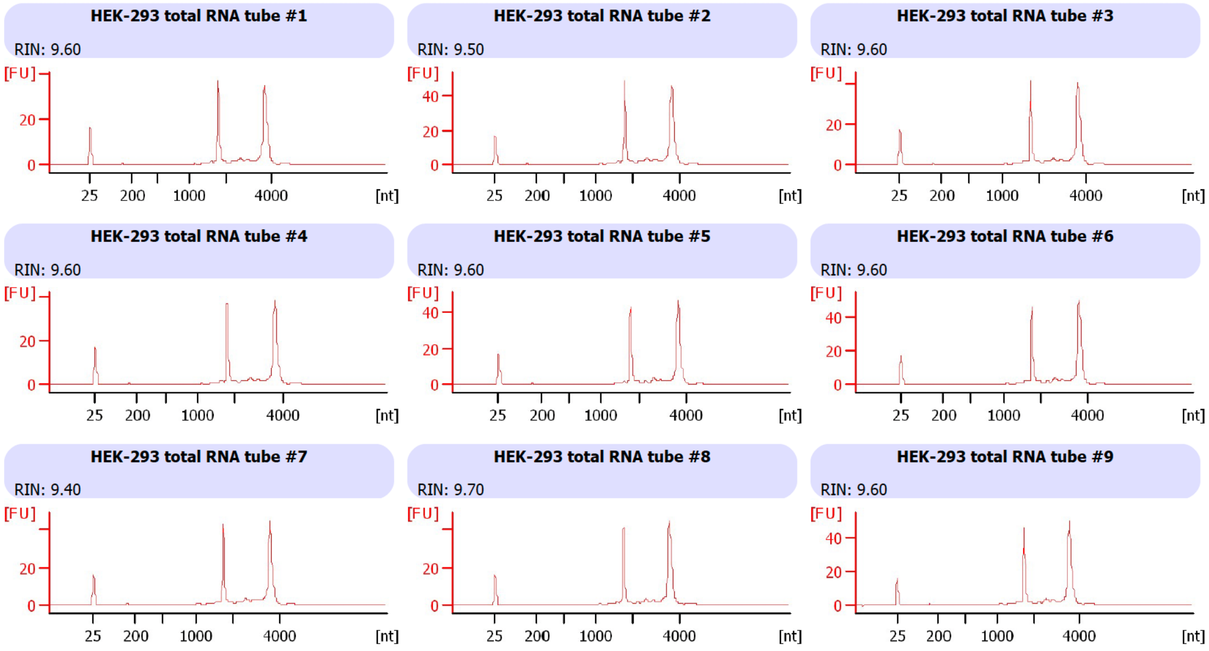

Supplementary Figure S1: Agilent Bioanalyzer tracings for the 9 preparations of total RNA from HEK293 cells at UM. See Methods for detailed descriptions of the cell growth, RNA isolation, and Bioanalyzer analyses.

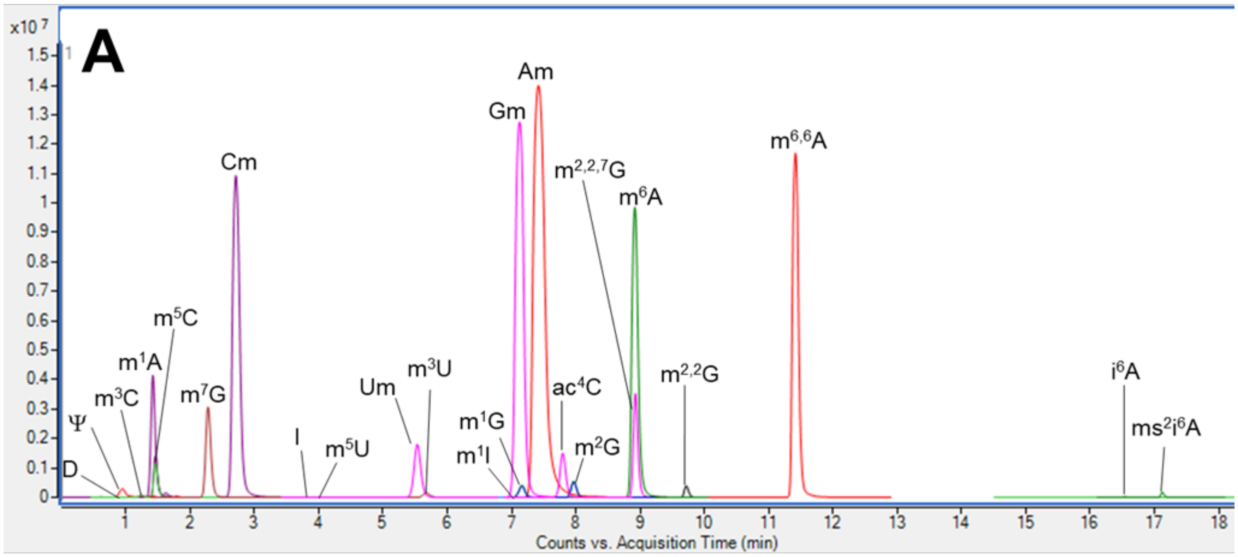

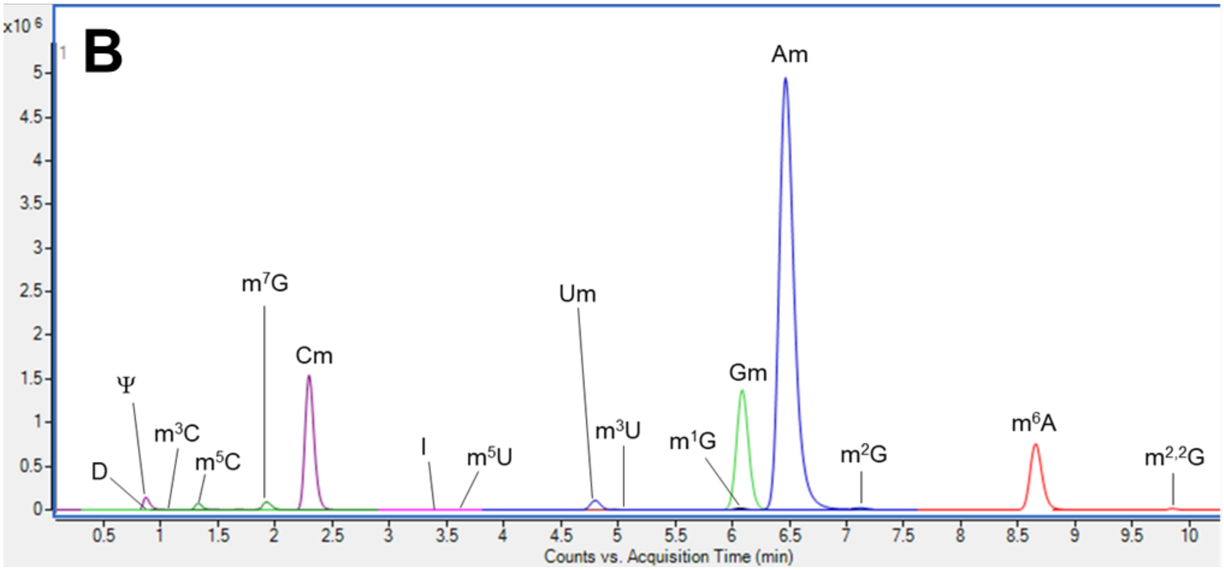

Supplementary Figure S2: Representative total extracted ion chromatograms from (**A**) relative and (**B**) absolute quantification of ribonucleosides by LC-MS by the MIT lab. Panel **A** relates the 23 ribonucleosides detected in samples analyzed in **Figure 3A**. Panel **B** relates to the 16 modifications analyzed in samples in **Figure 5**.

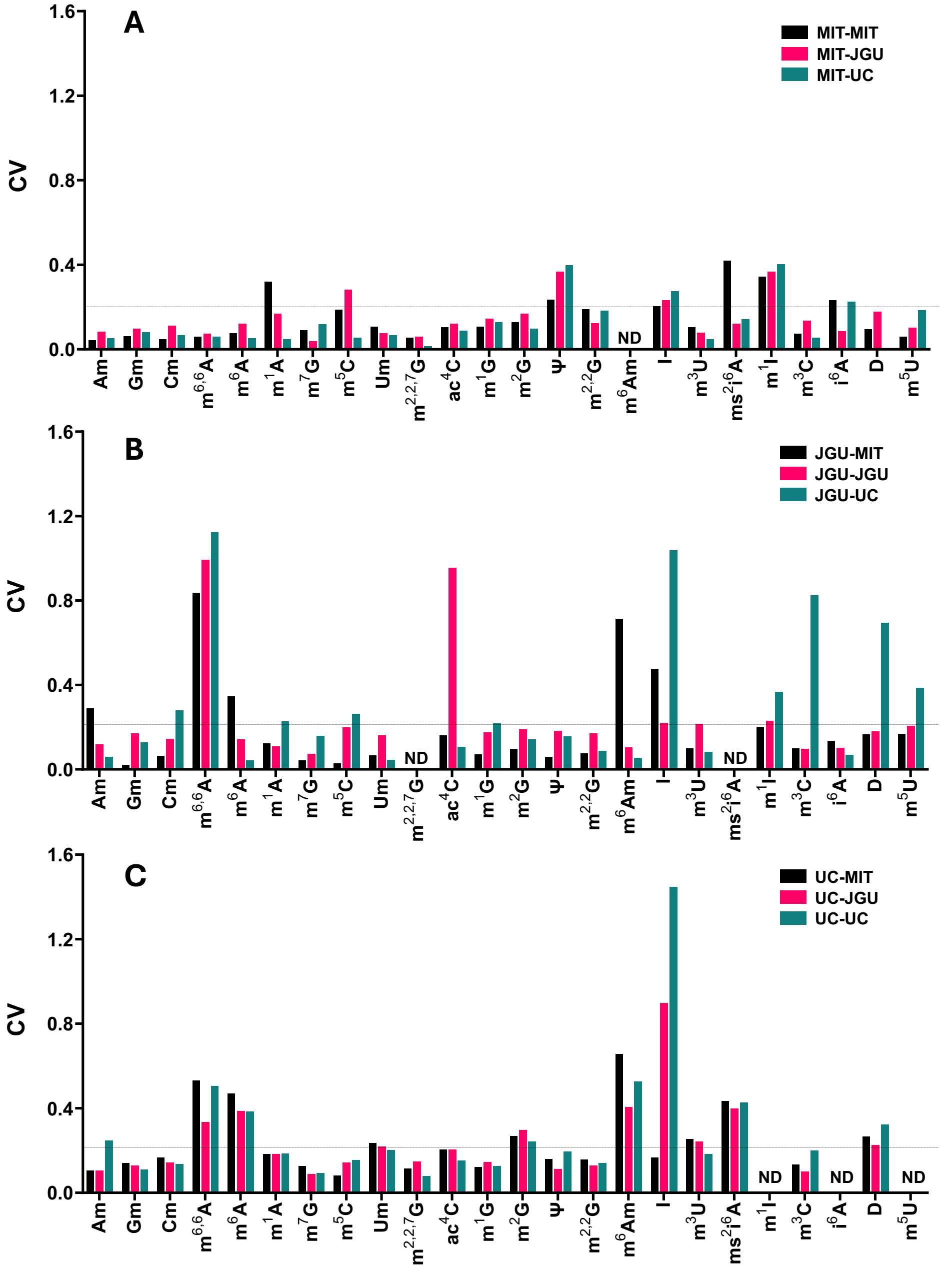

Supplementary Figure S3: Coefficient of variation (CV) results for normalized signal intensities obtained from each lab performing all three protocols. Data represent the mean CV for each lab (**A**, MIT; **B**, JGU; **C**, UC) performing each protocol (black, MIT; red, JGU, green, UC) in three replicate analyses. The dotted line denotes a CV of 20% as an approximation of a benchmark variance from three experienced labs and which other labs can expect to achieve.

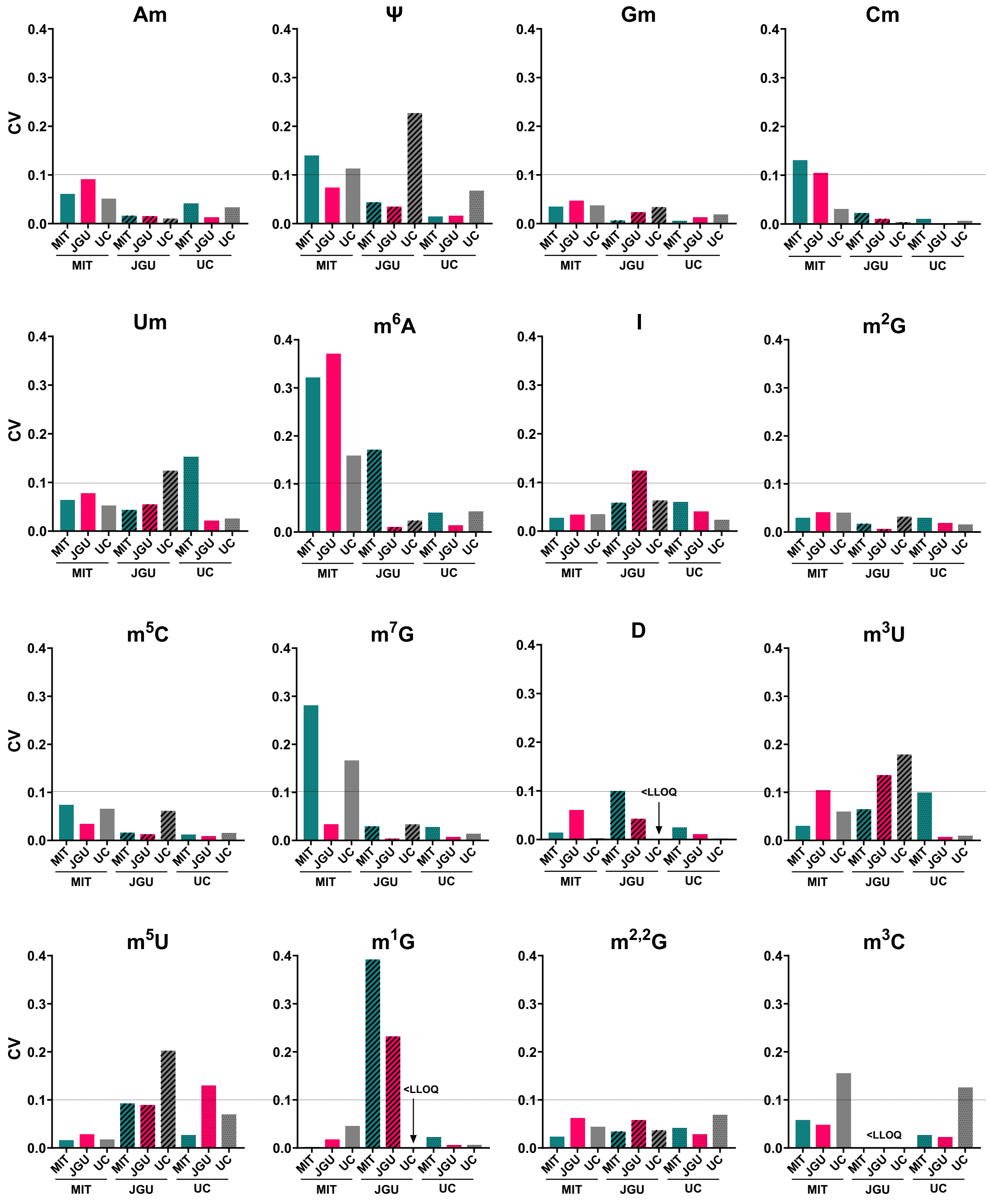

Supplementary Figure S4. Coefficient of variation (CV) results for absolute quantification of ribonucleosides by each lab using each of the three protocols. The black line denotes CV of 10%.

Supplementary Table S1: Sources of materials and reagents

UM = University of Michigan, JGU = Johannes Gutenberg University, MIT = Massachusetts Institute of Technology, UC = University of Cincinnati

(A) Cell culture and RNA isolation

	Reagents	UM
1	DMEM, high glucose, pyruvate	Fisher, 11-995-065
2	Fetal bovine serum	Biowest, S1480
3	Penicillin-Streptomycin	Fisher, 15-140-122
4	Phosphate buffered saline pH 7.4	Fisher, 10-010-049
5	Trypsin-EDTA (0.05%)	Fisher, 25-300-062
6	RPMI 1640 Medium	Fisher, 11-875-119
7	TRIzol Reagent	ThermoFisher, 15596026
8	Chloroform	VWR, 97064-678
9	Ethyl alcohol	MilliporeSigma, E7023
10	DEPC-Treated Water	ThermoFisher, AM9915G
11	NEB Next High Input Poly(A) mRNA Isolation Module	New England Biolabs, E3370S
12	RNeasy Midi Kit	Qiagen, 75144
13	2-Mercaptoethanol	MilliporeSigma, M3148
14	Ammonium Acetate	ThermoFisher, AM9070G
15	Lithium chloride	MilliporeSigma, L7026

(B) RNA hydrolysis

	Reagent	JGU	MIT	UC
1	Zinc chloride	Carl Roth		ACROS ORGANICS CAS-7646-85-7
2	Deferoxamine mesylate salt	Thermo Scientific	Sigma_D9533-1G	Sigma-Aldrich D9533-1G
3	Adenosine Deaminase inhibitor	Sigma-Aldrich		EMD-Millipore 116860-10MG
4	Butylated hydroxytoluene	Sigma-Aldrich/Merck	Sigma_W218405-1KG-K	Sigma-Aldrich PHR1117-1G /W218405-1KG-K
5	Phosphodiesterase I	Worthington Biochemical	Sigma_P3243_1VL	Fisher Scientific LS003926
6	Nuclease P1 from penicillium citrinum	Sigma-Aldrich		Sigma-Aldrich N8630-1VL
17	Thermo Scientific FastAp	Thermo Scientific		Thermo Scientific EF0651
8	Benzonase	Merck-Millipore	Sigma_70664-3	EMD-Millipore 70664-3

9	Phosphatase, Alkaline from bovine intestine	Sigma-Aldrich	Sigma_P5521_10KU	Sigma-Aldrich P7640-500MG / P5521-10KU
10	Magnesium chloride	Fisher Scientific (Invitogen)	Sigma_M8266-100G	
11	Tris-HCl, pH 8	Fisher Scientific (Invitogen)	Invitrogen 15568-025	
12	Coformycin		US National Cancer Institute Drug Repository	
13	Tetrahydrouridine	Merck-Millipore		
14	5-Bromocytidine			Ambeed Inc. A166879-100mg

(C) LC-MS/MS reagents

	Reagent	JGU	MIT	UC
1	Ammonium acetate	Sigma-Aldrich		Sigma-Aldrich 431311-250G
2	Acetic acid	Sigma-Aldrich		Fisher Scientific A11350
3	Acetonitrile	VWR	Sigma 34998-4L	Thermo Fisher Scientific A955-4
4	Formic acid		Thermo Sci. T85178-AD	

Supplementary Table S4: Four conditions for preparing RNA for shipping, prior to shipping either at ambient (room) temperature or on dry ice.

1	Shipped in 0.5 M NH_4OAc and 3 volumes EtOH
2	RNA was precipitated in 0.5 M $NH_4OAc$and 3 volumes EtOH; the resulting RNA pellet was air-dried and shipped
3	RNA was precipitated in 0.8 M LiCl and 3 volumes EtOH; the RNA pellet was air-dried and shipped
4	DEPC-treated water
